# Comprehensive Transcriptomic Analysis Identifies *ST8SIA1* as a Survival-Related Sialyltransferase Gene in Breast Cancer

**DOI:** 10.3390/genes11121436

**Published:** 2020-11-28

**Authors:** Jung-Yu Kan, Sin-Hua Moi, Wen-Chun Hung, Ming-Feng Hou, Fang-Ming Chen, Shen-Liang Shih, Jun-Ping Shiau, Chung-Liang Li, Chih-Po Chiang

**Affiliations:** 1 Department of Surgery, Kaohsiung Medical University Hospital, Kaohsiung 80756, Taiwan; kan890043@gmail.com (J.-Y.K.); mifeho@kmu.edu.tw (M.-F.H.); fchen@kmu.edu.tw (F.-M.C.); slshih1@gmail.com (S.-L.S.); gp5066@gmail.com (J.-P.S.); kmuhduty@gmail.com (C.-L.L.); 2 Division of Breast Surgery, Department of Surgery, Kaohsiung Medical University Hospital, Kaohsiung 80756, Taiwan; 3 Center of Cancer Program Development, E-Da Cancer Hospital, I-Shou University, Kaohsiung 82445, Taiwan; moi9009@gmail.com; 4 National Institute of Cancer Research, National Health Research Institutes, Tainan 70456, Taiwan; hung1228@nhri.edu.tw; 5 School of Pharmacy, College of Pharmacy, Kaohsiung Medical University, Kaohsiung 80708, Taiwan; 6 Drug Development and Value Creation Research Center, Kaohsiung Medical University, Kaohsiung 80708, Taiwan; 7 Graduate Institute of Clinical Medicine, Kaohsiung Medical University, Kaohsiung 80756, Taiwan; 8 Department of Medical Laboratory Sciences and Biotechnology, Fooyin University, Kaohsiung 83102, Taiwan

**Keywords:** hypersialylation, sialyltransferase, sialic acid, breast cancer, RNA sequencing, *ST8SIA1*

## Abstract

Hypersialylation caused by the overexpression of sialyltransferases (STs) is a common feature in cancer that is associated with several characteristics of tumorigenesis. Thus, identifying cancer-associated STs is critical for cancer therapy. However, ST screening has been frequently conducted in cell line models. In this study, we conducted a comprehensive analysis of STs in the clinical database and identified the STs related with the survival of breast cancer patients. RNA sequencing (RNA-Seq) data of 496 patients were obtained from The Cancer Genome Atlas Breast Invasive Carcinoma (TCGA-BRCA). Of the eight mapped STs, *ST3GAL5*, and *ST8SIA1* met the acceptable area under the curve (AUC) criteria for overall survival (OS). Using Kaplan–Meier methods, we determined that high expression of *ST8SIA1* was associated with poor 10-year OS in all patients, triple-negative breast cancer (TNBC), and non-TNBC patients, and poor disease-free survival (DFS) rates particularly in TNBC. *ST8SIA1* also had superior AUC values in terms of OS/DFS. High *ST8SIA1* levels showed a higher risk for poor OS in different groups of patients and a higher risk for poor DFS particularly in TNBC. In summary, we conducted a comprehensive analysis of STs from the clinical database and identified *ST8SIA1* as a crucial survival-related ST, which might be a potential therapeutic target for breast cancer and TNBC patients.

## 1. Introduction

Glycosylation is the main post-translational modification (PTM) in which glycans are conjugated to a protein at asparagine (N-linked) or serine/threonine (O-linked) residue, contributing to protein functions such as cell-to-cell interaction, recognition, adhesion, and migration. Aberrant glycosylation is a dominant feature of all human cancers that influences tumor proliferation, invasion, and metastasis. Of the glycosylation aberrations, hypersialylation is the most widely occurring cancer-associated glycosylation [1].

Sialic acid is a family of nine-carbon backbone glycans attached to the terminal position of glycoprotein/glycolipid on the cell surface. The process of sialic acid incorporation into glycoconjugates on the cell surface is known as sialylation, and it is catalyzed by sialyltransferases (STs). 20 STs have been categorized into four groups: ST3Gal1-5, ST6Gal1-2, ST6GalNAc1-6, and ST8SIA1-6. The four groups of STs are categorized according to (1) the linkage of carbon connecting the second carbon (C2) to the C3, C6, C8, and C9 positions, catalyzing the synthesis of α2,3-, α2,6-, α2,8-, and α2,9-linked sialic acids, and (2) the glycan substrate including Gal: sialic acid attached to the terminal position of galactose, GalNAc: sialic acid attached to the terminal position of N-acetyl galactose, and SIA: sialic acid attached to the terminal position of sialic acid [1,2,3].

Sialylation is crucial for cell-to-cell interaction, recognition, and adhesion. Desialylation is the primary physiological function of sialic acid. For instance, desialylation of serum glycoproteins and aging red blood cells are absorbed by hepatocytes and are recognized and degraded by macrophages [2,4,5]. On the contrary, hypersialylation on the cell surface gives malignant cells better survival, and this aberrant sialylation is accompanied by overexpression of STs. Aberrant sialylation has been well recognized in various human cancers with abnormal ST activity. For instance, ST6Gal1 has been reported to be involved in different aspects of tumorigenesis [6]. Oncogenic Ras can increase ST6Gal1 and alter sialylation of integrin β1 and the adhesion of cancer cells [7]. Sialylation of endothelial growth factor receptor (EGFR) was mediated by ST6Gal1 via the PI3K/Akt pathway to affect tumor proliferation [8,9]. In addition, sialylation of the Fas receptor (FasR) by ST6Gal1 has been shown to inhibit apoptotic signaling in colon cancer [10]. In summary, hypersialylation by α2,3-, α2,6-, and α2,8-STs could significantly affect tumor proliferation, adhesion, metastasis, apoptosis, immune evasion, and angiogenesis [11,12,13,14].

In breast cancer, STs were reported to be associated with stage/progression in 1980 [15]. Various α2,3-, α2,6-, and α2,8-STs were involved in breast cancer. For example, α2,3-STs have been shown to catalyze the sialylation of chemokine (C-C motif) receptor 7 (CCR7) to affect proliferation, invasion, and anoikis in breast cancer cells [16]. The adhesion and invasion abilities of breast cancer cells have been shown to be affected by α2,6-STs [17]. In addition, α2,8-STs were involved in tumor growth and metastasis of triple-negative breast cancer (TNBC) [18]. However, there is still some controversy about the role of STs in breast cancer. There was little agreement on the percentage of STs in breast cancer measured using immunohistochemistry [19]. Some α2,6-STs have been reported to function as metastasis suppressors in breast cancer [20], and intriguingly, sialic acid production in an advanced TNBC cell line was lower than that in an early stage cell line [21]. Comprehensive analysis of STs has only been conducted in cell line models [21,22,23], and there has been little agreement about STs measured using immunohistochemistry [19]. Thus, in this study, we aimed to investigate the expression of STs in breast cancer using RNA sequencing (RNA-Seq) data from The Cancer Genome Atlas Breast Invasive Carcinoma (TCGA-BRCA) of the Genomic Data Commons (GDC) data portal. We comprehensively analyzed expression of STs and its relation to the 10-year overall survival (OS) and disease-free survival (DFS) rates, which confirmed the importance of candidate STs and provided another therapeutic approach for breast cancer.

## 2. Materials and Methods

### 2.1. Study Population

The baseline characteristics and RNA-Seq expression data of selected genes were obtained from The Cancer Genome Atlas Breast Invasive Carcinoma (TCGA-BRCA) of the Genomic Data Commons (GDC) data portal (https://portal.gdc.cancer.gov/). Early stage intraductal carcinoma breast cancer patients were also included in the analysis. A total of 496 patients with comprehensive baseline characteristics and RNA-Seq expression data were analyzed. The baseline characteristics included age, ethnicity, molecular subtypes, pathological stage, lymph node invasion status, radiation, and pharmaceutical treatments. The included patients were divided into triple-negative breast cancer (TNBC) and non-triple-negative breast cancer (non-TNBC), according to the molecular subtypes of breast cancer. Overall survival (OS) was considered as a primary endpoint and disease-free survival (DFS) was considered as a surrogate endpoint.

### 2.2. RNA Sequencing Analysis

A total of 20 STs genes were included in the initial analysis. Only 8 STs genes were mapped by the RNA-Seq expression. Therefore, we estimated the different RNA-Seq expression levels regarding 8 STs genes between TNBC and non-TNBC groups, and those with significant differences between the subgroups were selected as target genes for later discriminant analysis. Level 3 data were downloaded from the TCGA data coordination center. The gene expression profile was determined experimentally using the Illumina HiSeq 2000 RNA Sequencing platform at the University of North Carolina TCGA genome characterization center. We conducted a differential gene expression (DGE) analysis to obtain standardized reading count data and conducted a statistical analysis to identify quantitative changes in gene expression levels based on RNA sequencing data. RNA-Seq expression was reported in reads per kilobase million (RPKM), and the current data set shows the gene-level transcription estimates as log2(x + 1)-transformed RSEM (RNA-Seq by Expectation-Maximization) normalized count.

### 2.3. Statistical Analysis

The baseline characteristics of the study population were presented as mean (±standard deviation, SD) or frequency (percentages). The difference in distribution between the TNBC and non-TNBC groups was estimated using the independent two-sample *t*-test, chi-squared test, or Fisher’s exact test. The RNA-Seq expression of genes in all patients was illustrated using a heatmap. The RNA-Seq expression in the entire study population, the TNBC group, and the non-TNBC group was expressed as mean ± SD, and the comparison of gene expression levels between TNBC and non-TNBC was evaluated using the independent two-sample *t*-test. Genes with statistically significant differences in RNA-Seq expression levels between TNBC and non-TNBC were chosen as target genes. Furthermore, receiver operating characteristic (ROC) analysis was used to estimate the optimal cutoff point of RNA-Seq expression for each target gene according to the OS and DFS status (death was set as an outcome event). All cut points were compared using the area under the curve (AUC), and an AUC greater than 0.6 was considered as an acceptable cutoff point. The patients in the entire study population, the TNBC group, and the non-TNBC group were divided into low and high expression levels according to the optimal cutoff points. The survival difference between low and high expression levels was illustrated using the Kaplan–Meier estimator and tested using the log-rank test. Univariate and multivariate Cox proportional hazard regression models were used to estimate the impact of RNA-Seq expression levels and baseline characteristics on OS and DFS in breast cancer. Hazard ratio (HR) and 95% confidence interval (CI) were computed. Moreover, differential expression analysis was conducted for the target genes with acceptable prediction ability for OS and PFS. All *p*-values were two-sided, and the statistical significance level for all tests was set at 0.05. All statistical analyses were performed using the computing environment R 4.0.2 (R Core Team, 2020, Vienna, Austria).

### 2.4. Gene Set Enrichment Analysis (GSEA) and Gene Ontology (GO)

Gene Set Enrichment Analysis (GSEA) is a computational approach that enables one to determine whether an a priori defined set of candidate genes shows statistically significant, concordant differences between two biological states or phenotypes. GSEA was conducted using the target genes to further explore related Gene Ontology (including BP: biological process, CC: cellular components, and MF: molecular functions) or enrichment pathways. The analysis was performed using TCGAbiolinks packages in R software. The comprehensive set of efficient and concise annotation tools were derived from the Database for Annotation, Visualization and Integrated Discovery (DAVID) [24]. The cutoff criterion for the GSEA was set at a false discovery rate (FDR) < 0.05.

## 3. Results

### 3.1. Characteristics of the Study Subjects from TCGA-BRCA

In this study, 496 samples were collected from TCGA-BRCA of the GDC data portal. These breast cancer samples were further divided into the triple-negative breast cancer (TNBC, *n* = 110) and non-TNBC (*n* = 386) groups. Baseline characteristics of the study population group by TNBC and non-TNBC are summarized in Table 1. A total of 110 TNBC patients with a mean age of 55.8 ± 12.4 years and 386 non-TNBC patients with a mean age of 56.9 ± 13.1 years were analyzed. Most of the patients were not Hispanic or Latino. There were significant differences in the distribution of the proportion of the pathological stage (*p* = 0.013) and lymph node invasion (*p* < 0.001) between TNBC and non-TNBC.

### 3.2. The Difference in RNA-Seq Expression of Sialyltransferase Genes

**Figure 1:**
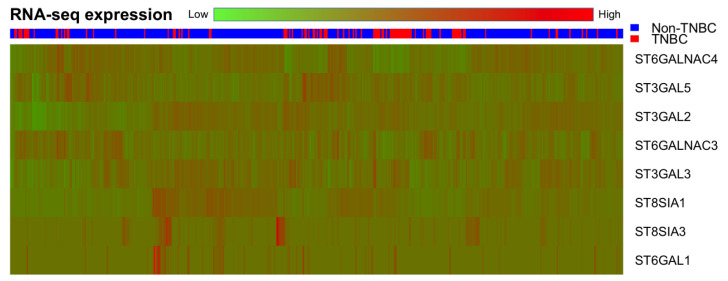
Heat map of RNA-Seq expression of the sialyltransferase genes. *ST3GAL5*, *ST6GALNAC4*, and *ST8SIA1* were candidate RNA-Seq expression target genes in breast cancer.

To verify the expression of different sialyltransferase genes in breast cancer, RNA-Seq expression of different sialyltransferase genes between TNBC and non-TNBC patients was calculated by reads per kilobase per million mapped reads (RPKM) and is summarized in Table 2. A total of 20 STs genes were included in the initial evaluation. Only 8 STs genes were mapped by the RNA-Seq expression. Overall, *ST3GAL5* had significantly higher RNA-Seq expression levels in the TNBC group (TNBC vs. non-TNBC: 3.86 ± 0.35 vs. 3.62 ± 0.30, *p* < 0.001). However, *ST6GALNAC4* (TNBC vs. non-TNBC: 3.66 ± 0.47 vs. 4.16 ± 0.36, *p* < 0.001) and *ST8SIA1* (TNBC vs. non-TNBC: 0.75 ± 0.76 vs. 1.04 ± 0.87, *p* < 0.001) had significantly higher RNA-Seq expression levels in the non-TNBC group. The heatmap of RNA-Seq expression of *ST3GAL2*, *ST3GAL3*, *ST3GAL5*, *ST6GAL1*, *ST6GALNAC3*, *ST6GALNAC4*, *ST8SIA1*, and *ST8SIA3* in each patient is illustrated in Figure 1. In the horizontal scale bar, the green color indicates a lower level of RNA-Seq expression and the red color indicates a higher level of RNA-Seq expression. Thus, *ST3GAL5*, *ST6GALNAC4*, and *ST8SIA1* were candidate genes for differential expression between TNBC and non-TNBC patients.

### 3.3. Distinction of OS by Sialyltransferase Gene in Breast Cancer

From the RNA-Seq expression results, *ST3GAL5*, *ST6GALNAC4*, and *ST8SIA1* were the candidate genes that showed statistically significant differential expression between TNBC and non-TNBC patients. Thus, we utilized OS status as the outcome to further determine the cutoff point and the ability of these candidate genes to discriminate OS and DFS. The ROC analysis results are shown in Table 3. ROC analysis was conducted to obtain the optimal cutoff point of RNA-Seq expression of *ST3GAL5*, *ST6GALNAC4*, and *ST8SIA1* according to the primary endpoint of OS status and the surrogate endpoint of DFS to confirm their importance. The ROC analysis results indicated that the optimal cutoff points of OS for *ST3GAL5*, *ST6GALNAC4*, and *ST8SIA1* were 3.63, 4.01, and 1.31, respectively. *ST8SIA1* (AUC = 0.68, sensitivity = 0.71, specificity = 0.73) showed the best predictive performance, followed by *ST3GAL5* (AUC = 0.62, sensitivity = 0.51, specificity = 0.71) and *ST6GALNAC4* (AUC = 0.54, sensitivity = 0.71, specificity = 0.44). However, only *ST3GAL5* and *ST8SIA1* met the acceptable criteria (AUC > 0.6).

### 3.4. High ST8SIA1 Expression Was Correlated with Poor OS/DFS in TNBC Patients

**Figure 2:**
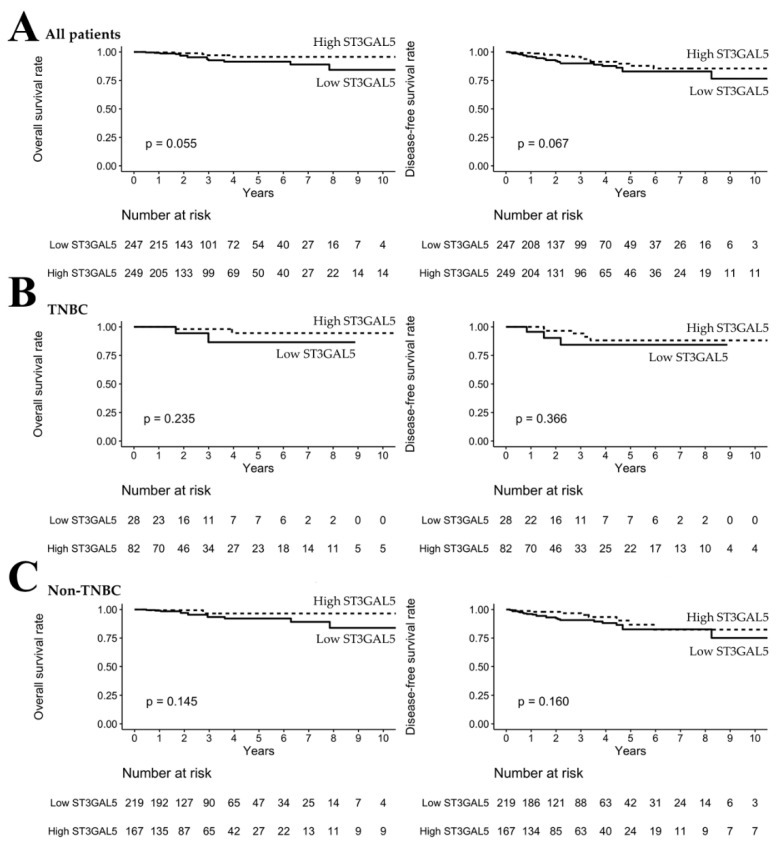
Kaplan–Meier analysis of *ST3GAL5* in breast cancer. (**A**–**C**) There was no statistically significant difference in the expression of *ST3GAL5* in all patients, triple-negative breast cancer (TNBC), and non-TNBC patients to discriminate overall survival (OS)/disease-free survival (DFS) status.

**Figure 3:**
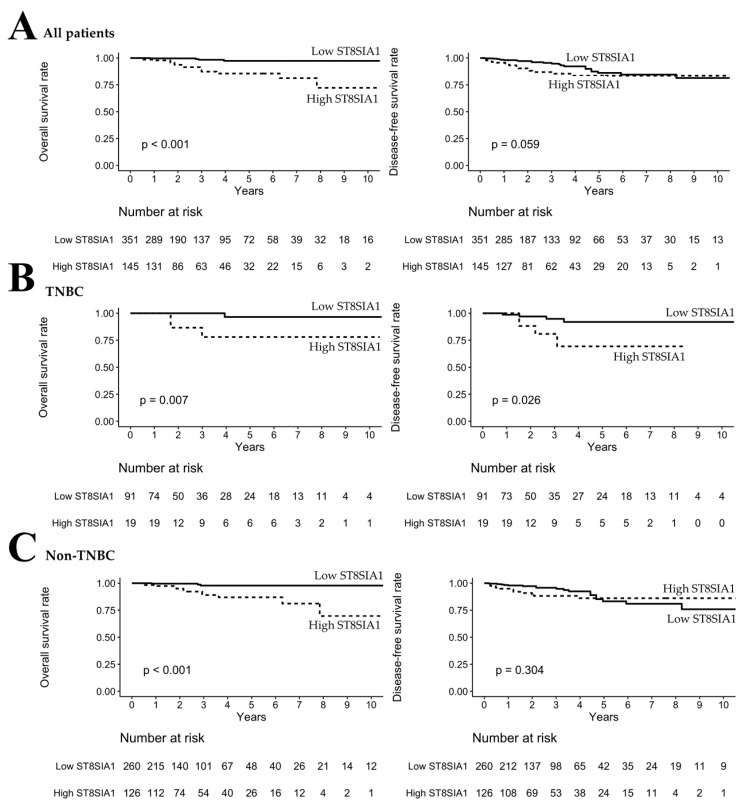
Kaplan–Meier analysis of *ST8SIA1* in breast cancer. (**A**,**C**) High expression of *ST8SIA1* correlated significantly with poor overall survival (OS) in all patients and non-triple negative breast cancer (TNBC) patients. (**B**) High expression of *ST8SIA1* correlated with poor OS and disease-free survival (DFS) in TNBC patients.

The study population was divided into high and low *ST3GAL5* and *ST8SIA1* RNA-Seq expression levels according to the optimal cutoff point of each gene. The OS and DFS analyses of *ST3GAL5* and *ST8SIA1* RNA-Seq expression levels were evaluated using the Kaplan–Meier method and are illustrated in Figure 2 and Figure 3, respectively. To examine the impact on OS/DFS, the study population was divided into three groups: all patients, TNBC, and non-TNBC. There was no statistically significant difference in the expression of *ST3GAL5* in all patients, TNBC, and non-TNBC of OS/DFS status (Figure 2). However, high expression of *ST8SIA1* was statistically significantly correlated with poor OS in all (Figure 3A, *p* < 0.001) and non-TNBC patients (Figure 3C, *p* < 0.001). Moreover, high expression of *ST8SIA1* was associated with poor OS (*p* = 0.007) and DFS (*p* = 0.026) in TNBC patients (Figure 3B). ROC analysis was also conducted to verify the ability of *ST3GAL5* and *ST8SIA1* to discriminate OS/DFS of TNBC and non-TNBC patients. The Kaplan–Meier results showed that *ST8SIA1* had superior AUC values compared to *ST3GAL5* (Table 4) regarding the ability to discriminate OS in TNBC (AUC of *ST8SIA1* = 0.78), non-TNBC (AUC of *ST8SIA1* = 0.68), and DFS in TNBC (AUC of *ST8SIA1* = 0.74). Thus, *ST8SIA1* affects the survival of breast cancer patients, particularly those with TNBC.

### 3.5. Cox Proportional Hazard Regression Model of ST8SIA1

According to the Kaplan–Meier and ROC results, *ST8SIA1* RNA-Seq expression levels that reached statistically significant survival differences were further included in the Cox proportional hazard regression model to estimate the impact of *ST8SIA1* levels and baseline characteristics on OS and DFS in all patients, TNBC patients, and non-TNBC patients. The Cox proportional hazard regression analyses for OS and DFS are presented in Table 5 and Table 6, respectively. In Table 5, the univariate analysis demonstrated that patients with high *ST8SIA1* levels (HR = 8.57, 95% CI = 2.84, 25.9, *p* < 0.001) had a higher risk for poor OS compared to patients with low *ST8SIA1* levels in the all patients group. Multivariate analysis also indicated that patients with high *ST8SIA1* levels (HR = 9.95, 95% CI = 3.25, 30.50, *p* < 0.001), those aged over 50 years (HR = 3.49, 95% CI = 1.09, 11.1, *p* = 0.035), those at pathological stage II (HR = 14.4, 95% CI = 1.55, 134, *p* = 0.019), and those at stage III (HR = 20.40, 95% CI = 1.47, 283, *p* = 0.025) were at higher risk for poor OS.

The study population was further divided into TNBC and non-TNBC groups. In the univariate analysis of the TNBC group, high *ST8SIA1* levels still showed a higher risk for poor OS (HR = 11.9, 95% CI = 1.23, 114.00, *p* = 0.032) compared to patients with low *ST8SIA1* levels, whereas in the non-TNBC group, both univariate and multivariate analysis showed that high *ST8SIA1* levels were associated with higher risk for poor OS (univariate: HR = 8.01, 95% CI = 2.26, 28.40, *p* = 0.001; multivariate: HR = 11.30, 95% CI = 3.04, 41.90, *p* < 0.001). Furthermore, diagnosis age, pathological stage, and radiation also impacted OS (Table 5). Interestingly, regarding DFS (Table 6), high *ST8SIA1* levels were associated with a higher risk for poor DFS in both univariate (HR = 4.30, 95% CI = 1.07, 17.30, *p* = 0.04) and multivariate analysis (HR = 5.13, 95% CI = 1.03, 25.50, *p* = 0.046) only in the TNBC groups. Thus, high expression of *ST8SIA1* could increase the risk for poor OS in breast cancer patients and particularly increased the risk for poor DFS in TNBC patients.

### 3.6. Gene Ontology Analysis (GO) of Candidate ST8SIA1 Gene

**Figure 4:**
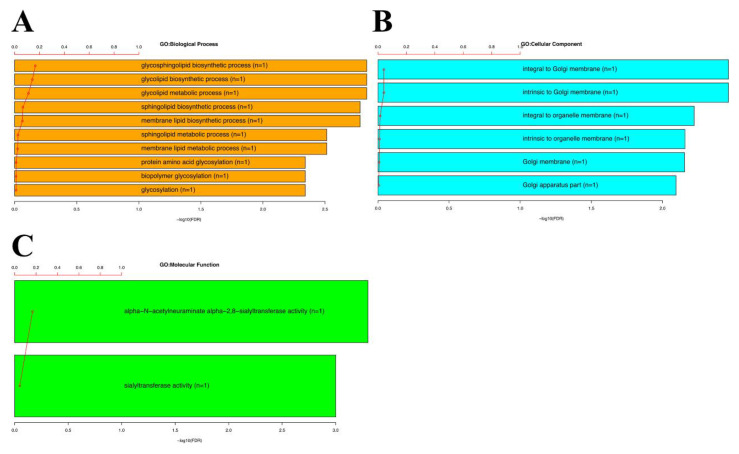
Gene Set Enrichment Analysis (GSEA) and Gene Ontology of *ST8SIA1.* (**A**–**C**) *ST8SIA1* was associated with the biological process of glycosylation, involved in the cellular components of the Golgi apparatus, and associated with the molecular functions of STs activity.

The survival-related STs of *ST8SIA1* were further analyzed through the GSEA of Gene Ontology analysis to determine the gene function (Figure 4). The Gene Ontology analysis included BP: biological process, CC: cellular components, and MF: molecular functions. Regarding BP, *ST8SIA1* was associated with intrinsic glycosphingolipid and glycosylation-related processes with a false discovery rate (FDR) of 0.001–0.004. Regarding CC, *ST8SIA1* was associated with organelle and Golgi membrane/apparatus (FDR = 0.001–0.008), which is the location of STs. Regarding MF, *ST8SIA1* was associated with intrinsic STs activity (FDR = 0.0004–0.0009). The results of GO analysis confirmed the genetic function of *ST8SIA1.*

## 4. Discussion

Hypersialylation is a feature of various cancers in which aberrant expression of STs increases sialylation expression on tumor cell surfaces. Hypersialylation further facilitates several aspects of tumorigenesis including (1) immune evasion through immune inhibitory Siglecs (Immune inhibitory receptors), (2) enhancement of tumor proliferation and metastasis through cytoskeleton-related protein, (3) promotion of tumor angiogenesis through the interaction between VEGF and polysialic acid, and (4) resistance to apoptosis through anti-apoptosis/kinase inhibitors [14]. The above effects of hypersialylation on cancer affect the survival of patients. Thus, it is crucial to elucidate survival-related STs in breast cancer and provide another therapeutic approach.

In our studies, we used RNA-Seq data from the clinical database of TCGA-BRCA of the GDC data portal to comprehensively analyze the role of STs in breast cancer. Prior to this study, a comprehensive analysis of STs had only been conducted in cell lines. In the cell line model, the author analyzed the transcript level of sialic acid metabolism and glycosylation (SAMG) genes including 20 STs in MCF10A, T-47D, and MDA-MB-231 cells [21,22]. In compartment 2 of 20 STs, MDA-MB-231 cells displayed higher levels of *ST3GAL5* and *ST8SIA1* than MCF10A/T-47D cells and no change was observed in *ST6GALNAC4.* Intriguingly, sialylation was lower in MDA-MB-231 than in T-47D cells due to metabolic flux-based control of sialylation [21,22]. In a proteomic analysis of different breast cancer cell lines, lectin chromatography/mass spectrometry was used to compare glycosylation profiles between luminal and TNBC cell lines. In contrast to the luminal type, a number of TNBC-specific glycosites were enriched with sialic acids and a concomitant increase in STs gene expression was observed [23]. The results of the proteomic analysis indicated that TNBC-specific sialylation might be a therapeutic target for this aggressive tumor subtype.

In our RNA-Seq analysis of the clinical data set, we identified three STs genes that were significantly different between TNBC and non-TNBC. *ST3GAL5* was higher in the TNBC group as in MDA-MB-231 cells, whereas *ST6GALNAC4* and *ST8SIA1* were higher in the non-TNBC group, which was somewhat different from the cell line model, indicating the difference of transcript level between cell line and clinical patients. In order to clarify the potential of STs as a therapeutic target, their effect on OS was investigated, and we found that *ST8SIA1* significantly impacted OS in all patients, the TNBC group, and the non-TNBC group. Moreover, *ST8SIA1* specifically affected DFS in TNBC patients, indicating that *ST8SIA1* might be a therapeutic target for this aggressive TNBC subtype.

*ST8SIA1*, also known as GD3 synthase gene (GD3S), is a key enzyme in the biosynthesis of b-/c-series gangliosides (GD3, GD2, and GT3) expressed on the cell surface. Gangliosides are glycosphingolipids carrying one or more sialic acid residues and are located on the outer leaflet of the plasma membrane to interact with receptor tyrosine kinases (RTKs) [25,26]. High expression of *ST8SIA1* or GD3S has been reported to be associated with poor histologic grade/survival in estrogen-receptor (ER)-negative breast cancer and with better prognosis in ER-positive breast cancer [27,28]. However, in our study, the impact of *ST8SIA1* on survival was not limited to ER-negative breast cancer. High expression of *ST8SIA1* was significantly correlated with poor OS in all patients, the TNBC group, and the non-TNBC group. In Cox proportional hazard regression, high expression of *ST8SIA1* was also significantly associated with higher risk of poor OS in all patients (HR = 8.57), the TNBC group (HR = 11.9), and the non-TNBC group (HR = 8.01). Thus, *ST8SIA1* is a crucial survival-related ST in breast cancer, regardless of the ER receptor status. Moreover, high expression of *ST8SIA1* was also significantly associated with poor DFS and a higher risk for poor DFS (HR = 4.30) in TNBC patients particularly. This TNBC-specific effect was similar to that found in other studies indicating an association between *ST8SIA1* and ER-negative breast cancer. The authors of several studies reported that *ST8SIA1* could promote tumor growth, metastasis, and chemoresistance through the FAK/Akt/mTOR and Wnt/β-catenin signaling pathways in TNBC cell lines [18,29,30]. This specific effect of *ST8SIA1* in TNBC cell lines may indicate the potential mechanisms underlying the association of higher *ST8SIA1* levels with poor DFS in TNBC patients. Thus, from the literature and our comprehensive study, survival-related STs of *ST8SIA1* may be a novel and crucial therapeutic target in breast cancer, especially for TNBC patients.

Several groups of sialyltransferase inhibitors (STIs) have been developed, including (1) analogs of sialic acids/CMP-sialic acid/cytidine/lithocholic acid, (2) oligosaccharide derivatives, (3) aromatic compounds, and (4) flavonoids [31,32]. Most STIs have poor cell membrane permeability and bioavailability that fail to meet the criteria for clinical utility. Few STIs have great permeability across the cell membrane. For instance, lithocholic acid analogs of AL10 exert great permeability and lower cytotoxicity, and they inhibit adhesion, migration, proliferation, and invasion of lung and breast cancer cells [11,16]. However, most STIs were developed against α2,3/2,6-STs, and fewer were developed to inhibit polysialyltransferases of α2,8-STs, especially *ST8SIA1.* Triptolide was isolated from the perennial vine *Tripterygium wilfordii* and displayed several anticancer cell properties [33,34]. Triptolide was also found to indirectly inhibit *ST8SIA1* in melanoma cells [35]. A specific STI against *ST8SIA1* is an unmet clinical need for breast cancer and TNBC treatment to address in a future study.

The present study still has some limitations. In our comprehensive transcriptomic analysis of STs from the TCGA-BRCA of the GDC data portal, 20 STs were included initially, but only 8 STs were mapped by the RNA-Seq expression. However, our studies have identified *ST8SIA1* as a crucial survival-related ST that is associated with higher risk for poor OS in all patients and DFS in TNBC patients particularly. Further in vitro and clinical studies are required to verify the expression and function of *ST8SIA1* beside the GO analysis and other members of STs to provide another therapeutic approach for breast cancer in the future.

## 5. Conclusions

In conclusion, we conducted a comprehensive transcriptomic analysis of STs using RNA-Seq data from the clinical TCGA-BRCA of the GDC data portal. With a different calculation approach, we identified *ST8SIA1* as a crucial survival-related ST. High expression of *ST8SIA1* displayed significant association with poor OS and indicated higher risk for poor OS in all breast cancer patients, TNBC, and non-TNBC patients. Furthermore, high expression of *ST8SIA1* exerted a significant association with poor DFS and higher risk for poor DFS in TNBC patients particularly. This study provides a valuable analysis of STs based on clinical patient data and indicates *ST8SIA1* as a potential therapeutic target for breast cancer and TNBC patients, particularly in the future.

## Figures and Tables

**Table 1:** Baseline demographics between triple-negative breast cancer (TNBC) and non-triple-negative breast cancer (non-TNBC) patients (*n* = 496).

Variables	Total, *n* = 496	TNBC, *n* = 110	Non-TNBC, *n* = 386	*p*
Age (years)	56.7 ± 13.0	55.8 ± 12.4	56.9 ± 13.1	0.390
Ethnicity				0.546
Hispanic or Latino	13 (2.6%)	3 (2.7%)	10 (2.6%)	
Not Hispanic or Latino	383 (77.2%)	89 (80.9%)	294 (76.2%)	
Not reported	100 (20.2%)	18 (16.4%)	82 (21.2%)	
Pathological stage				0.013
Stage I	95 (19.2%)	18 (16.4%)	77 (19.9%)	
Stage II	310 (62.5%)	81 (73.6%)	229 (59.3%)	
Stage III	91 (18.3%)	11 (10.0%)	80 (20.7%)	
Lymph node invasion	234 (47.2%)	40 (36.4%)	194 (50.3%)	<0.001
Treatment				
Radiation	274 (55.2%)	64 (58.2%)	210 (54.4%)	0.552
Pharmaceutical	410 (82.7%)	90 (81.8%)	320 (82.9%)	0.903
All-cause mortality (OS)	21 (4.2%)	4 (3.6%)	17 (4.4%)	0.933
Disease-progressed (PFI)	41 (8.3%)	8 (7.3%)	33 (8.5%)	0.816

*p*-value is estimated using an independent two-sample *t*-test, chi-squared test, or Fisher’s exact test.

**Table 2:** Comparison of RNA-Seq expression with reads per kilobase million (RPKM) of the selected gene between TNBC and non-TNBC samples.

Genes	Total	TNBC	Non-TNBC	*p*
ST3GAL2	2.79 ± 0.58	2.84 ± 0.66	2.78 ± 0.56	0.406
ST3GAL3	1.10 ± 0.30	1.12 ± 0.33	1.10 ± 0.29	0.638
ST3GAL5	3.67 ± 0.33	3.86 ± 0.35	3.62 ± 0.30	<0.001
ST6GAL1	0.02 ± 0.10	0.02 ± 0.06	0.03 ± 0.11	0.402
ST6GALNAC3	1.93 ± 0.20	1.95 ± 0.26	1.92 ± 0.18	0.177
ST6GALNAC4	4.05 ± 0.44	3.66 ± 0.47	4.16 ± 0.36	<0.001
ST8SIA1	0.98 ± 0.86	0.75 ± 0.76	1.04 ± 0.87	<0.001
ST8SIA3	0.16 ± 0.37	0.11 ± 0.27	0.17 ± 0.39	0.053

*p*-value is estimated using an independent two-sample *t*-test.

**Table 3:** Receiver operating characteristic (ROC) analysis for RNA-Seq expression of target genes according to survival status.

**Target Genes**	**OS**			
	**Optimal Cutoff Point**	**AUC**	**Sensitivity**	**Specificity**
ST3GAL5	≤3.63	0.62	0.51	0.71
ST6GALNAC4	≥4.01	0.54	0.71	0.44
ST8SIA1	≥1.31	0.68	0.71	0.73
	**DFS**			
	**Optimal Cutoff Point**	**AUC**	**Sensitivity**	**Specificity**
ST3GAL5	≤3.63	0.56	0.52	0.63
ST6GALNAC4	≥4.01	0.54	0.71	0.44
ST8SIA1	≥1.31	0.56	0.41	0.72

**Table 4:** ROC analysis for RNA-Seq expression of target genes according to survival status in TNBC and non-TNBC.

**Target Genes**	**OS**			
	**Optimal Cutoff Point**	**AUC**	**Sensitivity**	**Specificity**
TNBC ST3GAL5	≤3.63	0.64	0.75	0.50
ST6GALNAC4	≥4.01	0.67	0.22	0.75
ST8SIA1	≥1.31	0.78	0.75	0.85
Non-TNBC ST3GAL5	≤3.63	0.61	0.44	0.76
ST6GALNAC4	≥4.01	0.55	0.82	0.34
ST8SIA1	≥1.31	0.68	0.71	0.69
	**DFS**			
	**Optimal Cutoff Point**	**AUC**	**Sensitivity**	**Specificity**
TNBC ST3GAL5	≤3.63	0.52	0.63	0.25
ST6GALNAC4	≥4.01	0.50	0.22	0.75
ST8SIA1	≥1.31	0.74	0.50	0.85
Non-TNBC ST3GAL5	≤3.63	0.58	0.45	0.70
ST6GALNAC4	≥4.01	0.54	0.82	0.34
ST8SIA1	≥1.31	0.48	0.32	0.61

**Table 5:** Cox proportional hazard regression of overall survival (ST8SIA1) in all patients (*n* = 496).

Variables	Comparison	Univariate		Multivariate	
		HR (95% CI)	*p*	HR (95% CI)	*p*
**ST8SIA1**	High vs. low	8.57 (2.84, 25.9)	<0.001	9.95 (3.25, 30.5)	<0.001
Diagnosis age	≥50 vs. <50	2.88 (0.94, 8.89)	0.065	3.49 (1.09, 11.1)	0.035
Subtypes	TNBC vs. non-TNBC	0.77 (0.26, 2.29)	0.6	1.13 (0.36, 3.58)	0.8
Pathological stage	Stage II vs I	5.93 (0.78, 45.0)	0.085	14.4 (1.55, 134)	0.019
	Stage III vs I	5.46 (0.61, 49.3)	0.13	20.4 (1.47, 283)	0.025
Lymph node invasion	Positive vs. negative	1.45 (0.61, 3.48)	0.4	0.84 (0.30, 2.36)	0.7
Radiation	Yes vs. no	0.35 (0.13, 0.99)	0.047	0.22 (0.07, 0.69)	0.009
Pharmaceutical	Yes vs. no	0.64 (0.27, 1.51)	0.3	0.74 (0.30, 1.81)	0.5
Cox proportional hazard regression of overall survival (ST8SIA1) in TNBC patients (*n* = 110).					
**ST8SIA1**	High vs. low	11.9 (1.23, 114.00)	0.032	9.45 (0.78, 115.00)	0.078
Diagnosis age	≥50 vs. <50	0.75 (0.10, 5.30)	0.77	0.52 (0.07, 4.05)	0.5
Pathological stage	Stage III vs I	Omitted ^†^	-	Omitted ^†^	-
	Stage II vs I	Omitted ^†^	-	Omitted ^†^	-
Lymph node invasion	Positive vs. negative	5.66 (0.59, 54.50)	0.134	2.02 (0.13, 32.50)	0.6
Radiation	Yes vs. no	1.28 (0.13, 12.30)	0.831	1.2 (0.09, 16.00)	0.9
Pharmaceutical	Yes vs. no	Omitted ^†^	-	Omitted ^†^	-
^†^ Omitted as imbalanced of sample distribution.					
Cox proportional hazard regression of overall survival (ST8SIA1) in non-TNBC patients (*n* = 386).					
**ST8SIA1**	High vs. low	8.01 (2.26, 28.40)	0.001	11.30 (3.04, 41.90)	<0.001
Diagnosis age	≥50 vs.<50	4.96 (1.09, 22.60)	0.038	6.69 (1.42, 31.50)	0.016
Pathological stage	Stage III vs I	4.35 (0.56, 33.80)	0.16	15.50 (1.56, 154.00)	0.019
	Stage II vs I	4.12 (0.45, 37.40)	0.209	31.20 (1.99, 491.00)	0.014
Lymph node invasion	Positive vs. negative	0.93 (0.35, 2.53)	0.894	0.55 (0.16, 1.93)	0.4
Radiation	Yes vs. no	0.28 (0.09, 0.81)	0.019	0.16 (0.05, 0.56)	0.004
Pharmaceutical	Yes vs. no	0.52 (0.20, 1.35)	0.176	0.55 (0.19, 1.57)	0.3

**Table 6:** Cox proportional hazard regression of disease-free survival (ST8SIA1) in all patients (*n* = 496).

Variables	Comparison	Univariate		Multivariate	
		HR (95% CI)	*p*	HR (95% CI)	*p*
**ST8SIA1**	High vs. low	1.82 (0.97, 3.42)	0.063	1.82 (0.96, 3.45)	0.068
Diagnosis age	≥50 vs. <50	1.25 (0.65, 2.39)	0.5	1.37 (0.71, 2.66)	0.3
Subtypes	TNBC vs. non-TNBC	0.75 (0.34, 1.62)	0.5	0.85 (0.38, 1.90)	0.7
Pathological stage	Stage II vs I	2.12 (0.81, 5.54)	0.13	2.57 (0.89, 7.39)	0.081
	Stage III vs I	2.64 (0.88, 7.92)	0.083	3.32 (0.86, 12.9)	0.083
Lymph node invasion	Positive vs. negative	1.29 (0.70, 2.40)	0.4	0.85 (0.40, 1.82)	0.7
Radiation	Yes vs. no	0.96 (0.38, 2.47)	0.939	0.81 (0.31, 2.14)	0.7
Pharmaceutical	Yes vs. no	1.03 (0.54, 1.96)	0.921	1.05 (0.54, 2.04)	0.9
Cox proportional hazard regression of disease-free survival (ST8SIA1) in TNBC patients (*n* = 110).					
**ST8SIA1**	High vs. low	4.30 (1.07, 17.30)	0.04	5.13 (1.03, 25.50)	0.046
Diagnosis age	≥50 vs. <50	1.20 (0.29, 5.03)	0.804	0.98 (0.23, 4.21)	>0.9
Pathological stage	Stage III vs I	2.06 (0.25, 17.20)	0.506	1.51 (0.13, 17.70)	0.7
	Stage II vs I	3.70 (0.23, 60.00)	0.357	3.30 (0.10, 111.00)	0.5
Lymph node invasion	Positive vs. negative	3.07 (0.73, 12.90)	0.125	1.44 (0.22, 9.27)	0.7
Radiation	Yes vs. no	3.29 (0.40, 26.80)	0.266	3.31 (0.37, 29.90)	0.3
Pharmaceutical	Yes vs. no	Omitted ^†^	-	Omitted ^†^	
^†^ Omitted as imbalanced of sample distribution.					
Cox proportional hazard regression of disease-free survival (ST8SIA1) in non-TNBC patients (*n* = 386).					
**ST8SIA1**	High vs. low	1.45 (0.71, 2.94)	0.308	1.45 (0.71, 2.98)	0.3
Diagnosis age	≥50 vs. <50	1.19 (0.57, 2.47)	0.64	1.29 (0.61, 2.72)	0.5
Pathological stage	Stage III vs I	2.10 (0.71, 6.17)	0.179	3.05 (0.95, 9.77)	0.06
	Stage II vs I	2.43 (0.73, 8.11)	0.15	4.76 (1.05, 21.40)	0.042
Lymph node invasion	Positive vs. negative	0.95 (0.47, 1.90)	0.878	0.59 (0.24, 1.42)	0.2
Radiation	Yes vs. no	0.81 (0.31, 2.12)	0.674	0.67 (0.24, 1.81)	0.4
Pharmaceutical	Yes vs. no	0.88 (0.44, 1.76)	0.712	0.91 (0.44, 1.87)	0.8

## References

[1] Fuster M.M., Esko J.D. (2005). The sweet and sour of cancer: Glycans as novel therapeutic targets. Nat. Rev. Cancer.

[2] Schauer R. (2000). Achievements and challenges of sialic acid research. Glycoconj. J..

[3] Varki A. (2007). Glycan-based interactions involving vertebrate sialic-acid-recognizing proteins. Nat. Cell Biol..

[4] Ashwell G., Morell A.G. (2006). The Role of Surface Carbohydrates in the Hepatic Recognition and Transport of Circulating Glycoproteins. Adv. Enzymol. Relat. Areas Mol. Biol..

[5] Bratosin D., Mazurier J., Tissier J., Estaquier J., Huart J., Ameisen J., Aminoff D., Montreuil J. (1998). Cellular and molecular mechanisms of senescent erythrocyte phagocytosis by macrophages: A review. Biochimie.

[6] Garnham R., Scott E., Livermore K.E., Munkley J. (2019). ST6GAL1: A key player in cancer (Review). Oncol. Lett..

[7] Seales E.C., Shaikh F.M., Woodard-Grice A.V., Aggarwal P., McBrayer A.C., Hennessy K.M., Bellis S.L. (2005). A Protein Kinase C/Ras/ERK Signaling Pathway Activates Myeloid Fibronectin Receptors by Altering β1 Integrin Sialylation. J. Biol. Chem..

[8] Liu Q., Ma H., Sun X., Liu B., Xiao Y., Pan S., Zhou H., Dong W., Jia L. (2019). The regulatory ZFAS1/miR-150/ST6GAL1 crosstalk modulates sialylation of EGFR via PI3K/Akt pathway in T-cell acute lymphoblastic leukemia. J. Exp. Clin. Cancer Res..

[9] Chang T.-C., Chin Y.-T., Nana A.W., Wang S.-H., Liao Y.-M., Chen Y.-R., Shih Y.-J., Changou C.A., Yang Y.-C.S., Wang K. (2018). Enhancement by Nano-Diamino-Tetrac of Antiproliferative Action of Gefitinib on Colorectal Cancer Cells: Mediation by EGFR Sialylation and PI3K Activation. Horm. Cancer.

[10] Swindall A.F., Bellis S.L. (2011). Sialylation of the Fas Death Receptor by ST6Gal-I Provides Protection against Fas-mediated Apoptosis in Colon Carcinoma Cells. J. Biol. Chem..

[11] Chiang C.-H., Wang C.-H., Chang H.-C., More S.V., Li W.-S., Hung W.-C. (2010). A novel sialyltransferase inhibitor AL10 suppresses invasion and metastasis of lung cancer cells by inhibiting integrin-mediated signaling. J. Cell. Physiol..

[12] Li F., Ding J. (2019). Sialylation is involved in cell fate decision during development, reprogramming and cancer progression. Protein Cell.

[13] Chiodelli P., Urbinati C., Paiardi G., Monti E., Rusnati M. (2018). Sialic acid as a target for the development of novel antiangiogenic strategies. Futur. Med. Chem..

[14] Zhou X., Yang G., Guan F. (2020). Biological Functions and Analytical Strategies of Sialic Acids in Tumor. Cells.

[15] Ip C., Patel J., Dao T.L. (1980). Serum Sialyltransferase and 5′-Nucleotidase as Reliable Biomarkers in Women with Breast Cancer. J. Natl. Cancer Inst..

[16] Su M.-L., Chang T.-M., Chiang C.-P., Chang H.-C., Hou M.-F., Li W.-S., Hung W.-C. (2014). Inhibition of Chemokine (C-C Motif) Receptor 7 Sialylation Suppresses CCL19-Stimulated Proliferation, Invasion and Anti-Anoikis. PLoS ONE.

[17] Cheng J., Wang R., Zhong G., Chen X., Cheng Y., Li W., Yang Y. (2020). ST6GAL2 Downregulation Inhibits Cell Adhesion and Invasion and is Associated with Improved Patient Survival in Breast Cancer. OncoTargets Ther..

[18] Nguyen K., Yan Y., Yuan B., Dasgupta A., Sun J.C., Mu H., Do K.-A., Ueno N.T., Andreeff M., Battula V.L. (2018). ST8SIA1 Regulates Tumor Growth and Metastasis in TNBC by Activating the FAK–AKT–mTOR Signaling Pathway. Mol. Cancer Ther..

[19] Julien S., Videira P.A., Delannoy P. (2012). Sialyl-Tn in Cancer: (How) Did We Miss the Target?. Biomology.

[20] Murugaesu N., Iravani M., Van Weverwijk A., Ivetic A., Johnson D.A., Antonopoulos A., Fearns A., Jamal-Hanjani M., Sims D., Fenwick K. (2014). An In Vivo Functional Screen Identifies ST6GalNAc2 Sialyltransferase as a Breast Cancer Metastasis Suppressor. Cancer Discov..

[21] Saeui C.T., Nairn A.V., Galizzi M., Douville C., Gowda P., Park M., Dharmarha V., Shah S.R., Clarke A., Austin M. (2018). Integration of genetic and metabolic features related to sialic acid metabolism distinguishes human breast cell subtypes. PLoS ONE.

[22] Saeui C.T., Cho K.-C., Dharmarha V., Nairn A.V., Galizzi M., Shah S.R., Gowda P., Park M., Austin M., Clarke A. (2020). Cell Line-, Protein-, and Sialoglycosite-Specific Control of Flux-Based Sialylation in Human Breast Cells: Implications for Cancer Progression. Front. Chem..

[23] Drake P.M., Schilling B., Niles R.K., Prakobphol A., Li B., Jung K., Cho W., Braten M., Inerowicz H.D., Williams K. (2012). Lectin Chromatography/Mass Spectrometry Discovery Workflow Identifies Putative Biomarkers of Aggressive Breast Cancers. J. Proteome Res..

[24] Da Huang W., Sherman B.T., Lempicki R.A. (2009). Systematic and integrative analysis of large gene lists using DAVID bioinformatics resources. Nat. Protoc..

[25] Todeschini A.R., Hakomori S.-I. (2008). Functional role of glycosphingolipids and gangliosides in control of cell adhesion, motility, and growth, through glycosynaptic microdomains. Biochim. Biophys. Acta.

[26] Grouxdegroote S., Rodríguez-Walker M., Dewald J.H., Daniotti J.L., Delannoy P. (2018). Gangliosides in Cancer Cell Signaling. Prog. Mol. Biol. Transl. Sci..

[27] Ruckhäberle E., Rody A., Engels K., Gaetje R., Von Minckwitz G., Schiffmann S., Grösch S., Geisslinger G., Holtrich U., Karn T. (2008). Microarray analysis of altered sphingolipid metabolism reveals prognostic significance of sphingosine kinase 1 in breast cancer. Breast Cancer Res. Treat..

[28] Ruckhaeberle E., Karn T., Rody A., Hanker L., Gätje R., Metzler D., Holtrich U., Kaufmann M. (2009). Gene expression of ceramide kinase, galactosyl ceramide synthase and ganglioside GD3 synthase is associated with prognosis in breast cancer. J. Cancer Res. Clin. Oncol..

[29] Cazet A., Lefebvre J., Adriaenssens E., Julien S., Bobowski M., Grigoriadis A., Tutt A., Tulasne D., Le Bourhis X., Delannoy P. (2010). GD3 Synthase Expression Enhances Proliferation and Tumor Growth of MDA-MB-231 Breast Cancer Cells through c-Met Activation. Mol. Cancer Res..

[30] Wan H., Li Z., Wang H., Cai F., Wang L. (2020). ST8SIA1 inhibition sensitizes triple negative breast cancer to chemotherapy via suppressing Wnt/beta-catenin and FAK/Akt/mTOR. Clin. Transl. Oncol..

[31] Wang L., Liu Y., Wu L., Sun X.-L. (2016). Sialyltransferase inhibition and recent advances. Biochim. Biophys. Acta.

[32] Szabo R., Skropeta D. (2017). Advancement of Sialyltransferase Inhibitors: Therapeutic Challenges and Opportunities. Med. Res. Rev..

[33] Shamon L.A., Pezzuto J.M., Graves J.M., Mehta R.R., Wangcharoentrakul S., Sangsuwan R., Chaichana S., Tuchinda P., Cleason P., Reutrakul V. (1997). Evaluation of the mutagenic, cytotoxic, and antitumor potential of triptolide, a highly oxygenated diterpene isolated from Tripterygium wilfordii. Cancer Lett..

[34] Phillips P.A., Dudeja V., McCarroll J.A., Borja-Cacho D., Dawra R.K., Grizzle W.E., Vickers S.M., Saluja A.K. (2007). Triptolide Induces Pancreatic Cancer Cell Death via Inhibition of Heat Shock Protein. Cancer Res..

[35] Kwon H.-Y., Kim S.-J., Kim C.-H., Son S.-W., Kim K.-S., Lee J.-H., Do S.-I., Lee Y.-C. (2010). Triptolide downregulates human GD3 synthase (hST8Sia I) gene expression in SK-MEL-2 human melanoma cells. Exp. Mol. Med..

